# LINGUISTIC MEASURES OF SPOKEN UTTERANCES FOR DETECTING MILD COGNITIVE IMPAIRMENT

**DOI:** 10.1093/geroni/igz038.826

**Published:** 2019-11-08

**Authors:** Meysam Asgari, Jeffrey Kaye, Hiroko Dodge

**Affiliations:** Oregon Health & Science University, Portland, Oregon, United States

## Abstract

Studies have shown that speech characteristics can aid in early-identification of those with mild cognitive impairment (MCI). We performed a linguistic analysis on spoken utterances of 41 participants (15 MCI, 26 healthy controls) from conversations with a trained interviewer using the Term Frequency-Inverse Document Frequency (TF-IDF) method. Data came from a randomized controlled behavioral clinical trial (ClinicalTrials.gov: NCT01571427) to examine effects of conversation-based cognitive stimulation on cognitive functions among older adults with normal cognition or MCI, which served as a pilot study for I-CONECT. From the collected spoken utterances we first constructed a fixed-dimensional feature vector using TF-IDF. Next, to distinguish between MCI and healthy controls, we trained a support vector machine (SVM) classifier on per-subject feature vectors according to 5-fold cross-validation procedure. Our results verify the effectiveness of TF-IDF features in this classification task with Receiver Operating Characteristic Area Under Curve of 81%, well above chance at 65%.

